# Cow efficiency: modeling the biological and economic output of a Michigan beef herd

**DOI:** 10.1093/tas/txaa166

**Published:** 2020-09-10

**Authors:** Logan R Thompson, Matthew R Beck, Daniel D Buskirk, Jason E Rowntree, Melissa G S McKendree

**Affiliations:** 1 Department of Animal Science, Michigan State University, East Lansing, MI; 2 Faculty of Agriculture and Life Sciences, Lincoln University, Canterbury, New Zealand; 3 Department of Agricultural, Food, and Resource Economics, Michigan State University, East Lansing, MI

**Keywords:** beef, cow-calf, economics, efficiency, net present value

## Abstract

In recent decades, beef cattle producers have selected cattle for biological traits (i.e., improved growth) to maximize revenue, leading to an increase in average cow body size. However, matching cow size to the production environment would allow producers to maximize productivity and economic returns per unit of land. This may help meet the goals of sustainable intensification, but environmental complexity and varying cow-calf production systems dictates a regional approach. The objective of this experiment was to examine the biological efficiency and economic returns of a Northern Michigan cow-calf system. We hypothesized that biological efficiency and economic returns would decrease with increasing cow body size. Data were collected from a Red Angus cow herd located at the Lake City AgBio Research Center in Lake City, MI from 2011 to 2018 on cow age, weight, and body condition score at weaning, and subsequent 205 d adjusted calf weaning weight (WW), sex, and yearling weight. Biological efficiency was defined as WW as a percentage of cow body weight (DBW). Enterprise budgeting techniques were used to calculate expected net returns from 2011 to 2018 after classifying cows into 11 BW tiers at 22.67 kg intervals beginning at 430.83 kg. Forward-looking net present value (NPV) was calculated using the same tier system, for a 10-yr production cycle with the baseline being a 200 d grazing season. Weaning weight increased with increasing DBW (*P* < 0.01), but the percentage of cow body weight weaned was reduced by −38.58 × Ln(DBW) (*P* < 0.01). This led to cows weaning 26.38 kg/ha more with every 100 kg drop in DBW. Expected net returns from 2011 to 2018 did not differ by DBW tier on a per cow basis but did on a per ha basis with a decrease in $10.27/ha with each increase in DBW tier (*P* < 0.01). Net present value was maximized in the baseline scenario at 453.51 kg DBW and decreased in value as DBW increased. These results suggest that for a Northern Midwestern cow-calf herd, comparatively lighter cows provide a higher economic value on a land basis.

## INTRODUCTION

Matching cow size to their environment plays a key role in the long-term sustainability of the operation but selection for biological performance (i.e., average daily gain, kg/d) indicators may be disrupting this balance. This can be seen by the increase in cow size in recent decades (Johnson et al., 2010; Scasta et al., 2015). Producers choose growth traits in an effort to maximize revenue which has led to increases in mature cow size. Additionally, producers have been incentivized by packers to produce bigger, heavier carcasses (Johnson et al., 2010). However, this may be hampering the long-term environmental and economical sustainability of cow-calf operations if producers have to adjust stocking rates due to selecting for traits which provide short-term benefits (Miller et al., 2001; Doye and Lalman, 2011). In conjunction with these economic incentives, producers have a tendency to subconsciously favor larger animals because of the perceived benefits in growth, but they do not always consider the feed requirements of the herd are increasing and fail to adjust for herd size (Doye and Lalman, 2011; Reuter, 2017). Therefore, recommendations are required for producers to make informed decisions on suitable cow sizes for their specific environments. The objective of this study was to model the relationship between cow body size and age on calf weaning weight (WW), yearling performance, cow longevity, and economic returns of a Northern Michigan cow herd. We hypothesized that WW efficiency and economic returns would decrease with increasing cow body size.

Biological variables such as cow body weight and WW play an important role in production system efficiency. Dickerson (1970) defined efficiency as the ratio of total cost to total animal product and highlighted the need to maximize production per female relative to their metabolic body weight. Previous literature, including classical work by Urick et al. (1971) and Dinkel and Brown (1978), has shown that as cow body weight increases producers may realize moderate improvements in calf WW. Recent studies including Kuhlers et al. (2013), Mourer et al. (2010), and Dobbs et al. (2011) have shown that for each additional 100 kg of cow BW, calf WW may increase from 2.3 to 10 kg depending on stocking rate and environmental conditions. However, the ability of heavier cows to wean heavier calves may not come efficiently (Doye and Lalman, 2011; Scasta et al., 2015). Lalman and Beck (2019) reported that mature cow size has been increasing and requires increased income to offset the increase in mature cow size. Whitworth et al. (2006) examined the biological efficiency of the University of Arkansas beef herd and found the efficiency was lowest with heavier cows. This would imply that as cow size has increased their ability to wean more kilograms has been compromised.

Biological efficiency ratios are incomplete metrics for genetic selection criteria and are more useful when paired with an economic analysis. An important factor that influences biological efficiency and cattle performance is the maintenance overhead (energy intake required for maintenance) and this will vary by production environment (Arango and Van Vleck, 2002). Doye and Lalman (2011) performed an economic analysis of two cow herds in the southern plains of differing sizes (498.86 kg vs. 634.92 kg) and two pasture systems (native and improved pasture). They reported that larger cows weaned more weight and therefore generated more revenue from the sale of the calves. However, the greater nutritional costs, fewer cows per unit of land, and overall higher fixed cost per cow for heavier animals resulted in a decrease in returns compared to the lighter cows. Bir et al. (2018) later calculated the net present value (NPV) of increasingly heavier cows and found that 430.84 kg cows provided the highest return across different grazing scenarios and beef breeds.

The type of cow that optimizes a production system in one region may not in another, investigations into beef cow efficiency in Michigan and the Northern Midwest has yet to be done. Understanding the production efficiency of Michigan beef cows will provide insights for improving the regions sustainability as it relates to sustainable intensification. The concept of sustainable intensification implies the maximization of production per unit of land in a manner that meets all three pillars of sustainability—planet, people, and profit (Makkar, 2013). If lighter cows require less land to produce a similar amount of product and be as or more profitable, this would address two of the three pillars. However, sustainable intensification does not specify how to meet these goals and regional complexity means that different strategies will be needed depending on local climate, production systems, and management.

## MATERIALS AND METHODS

To examine both the biological and economic efficiency of cows of differing weights a multistep approach was conducted. Collected data were subject to statistical analysis to develop biological output models. Additionally, economic costs and prices were collected to conduct a backward-looking enterprise budget analysis of the cow herd. These biological output models, costs, and prices were then used in a forward-looking NPV analysis of modeled cow herds of increasing weight classes.

### Animals and Forage

Data for this study were obtained from the Lake City AgBioResearch Station cow herd from 2011 to 2018 in northwest Michigan (Lake City, MI). Annual precipitation from 2011 to 2018 was 86.90 cm, and average monthly high (°C) and low temperatures and precipitation over this period is detailed in Table 1 (usclimatedata.com) (U.S. Climate Data, 2020).

**Table 1.** Average climate Data Lake City, MI 2011–2018^*a*^

Month	High T, °C	Low T, °C	Precip., cm
January	−2.69	−12.19	4.82
February	−1.40	−12.24	3.78
March	4.64	−8.13	4.96
April	10.79	−1.76	11.09
May	20.20	5.98	8.69
June	24.56	10.64	7.32
July	27.63	13.04	8.68
August	26.11	12.17	7.55
September	22.52	7.76	7.32
October	14.46	2.39	10.81
November	6.44	−2.77	6.89
December	0.24	−7.30	5.00
Total	—	—	86.90

^*a*^usclimatedata.com.

The cow herd from 2011 to 2018 were Red Angus cows and resulted in a total of 1,038 cow-calf records, an average of 130 annual records. Only cows that produced a calf each year were recorded in the data set and reasons for culling were not recorded. Cows were managed in an adaptive multipaddock grazing system with improved forages including: orchardgrass (*Dactylis glomerate* L.), alfalfa (*Medicago Sativa* L.), timothy grass (*Phleum pretense*), red clover (*Trifolium pratense* L.), white clover (*Trifolium repens* L.), birdsfoot trefoil (*Lotus corniculatus* L.), smooth bromegrass (*Bromus inermis* L.), and Kentucky bluegrass (*Poa pratensis*). Grazing onset began when forage availability was determined to be adequate based on visual appraisal, with turn out averaging May 15th and grazing terminated due to snow cover by mid-November. From 2011 to 2018 the farm averaged approximately 200 grazing days per year. Cows were managed in a grazing system using high stock densities (~150,000 kg ha^−1^) until 2016 when management was shifted to an adaptive rotational system with larger paddocks and longer grazing durations with a target grazing density of ~80,000 kg/ha. Winter management consisted of high quality (9% to 11% crude protein) grass hay fed ab libitum. Hay was fed by rolling hay bales across fields and winter-feeding locations were rotated across the farm depending on where animal impact was desired. Cows received ad libitum water and offered free-choice mineral.

Bulls were introduced on approximately July 1 each year for heifers and at the end of July for mature cows, with calving occurring from late March to May. Calves were weaned in October and November (~6 mo of age) of each year. Each year at weaning cow body weights were recorded, and cow body condition score (BCS) was recorded on a 1 to 9 scale by two qualified technicians (Wagner et al., 1988), with the exception of 2018 when BCS was not recorded. Calf WW were taken and adjusted to a 205 d WW with no age of dam adjustment included (WW). Weaned calves went into a grass-finishing system described by Stanley et al. (2018) and yearling weights (YW) were recorded the spring after weaning. All mature cows were managed the same over the 8-yr period except for a small subset used for a grazing experiment which were removed from the analysis. Retained heifers were managed separately from mature cows and joined the mature cow herd after first calving. Cow body weights were then normalized to a BCS of 5 using equations described by Fox et al. (1988; DBW). For 2018, actual cow body weights were used due to lack of BCS records that year. Biological efficiency was calculated for both 205 d adjusted WW (CWP) and YW as a percentage of DBW.

### Forage Intake and Land Use

Calves were assumed to enter the finishing enterprise at weaning and were not included in forage intake or economic analyses. Forage intake for the cow-calf enterprise was calculated using NEm values of the forage base and fed hay as recommended by NASEM (2016; see Equation 1). Forage samples from 2012 and 2013 were analyzed by Dairy One (Ithica, NY) with sampling procedures described by Chiavegato et al. (2015) and hay core samples were taken from 2016 to 2018 and analyzed by DairyLand (Battle Creek, MI).^1^ Intake was calculated as:

$$\begin{array}{l} Dry\;Matter\;intake\;(DMI),Kg/d\\ \;=[SBW^{0.75}\times (0.04997\times NEm^{2}+0.04631)]/NEm \end{array}$$ (1)

Intake of nonpregnant cows included an intake adjustment of 0.0384 (NASEM, 2016). During lactation, cows were assumed to have a peak lactation of 8 kg/d at maturity and intake was increased by adding 0.2 × daily milk yield adjusted for age of cow (NASEM, 2016). Net energy maintenance values were 1.43 Mcal/kg dry matter (DM) for pasture and 1.20 Mcal/kg DM for hay. Yearly forage consumption per cow was estimated using a 200 d grazing season (Lake City Research Center Beef Report, 2014) and 164 d of hay consumption. During the hay feeding period, 115 d were modeled to include environmental stress (i.e., cold stress) with a 5% increase in DMI per day using the dry pregnant cow calculation (NRC, 2000; usclimatedata.com). Since the grazing season aligns with the farm calving season, intake was calculated using the lactating intake. Stocking rate was then calculated using on farm estimates of forage utilization rates from 2014 to 2018, based on cow days per acre and assuming an average forage utilization of 50%. For years falling outside of this range, the average (5506.18 kg/ha) was used.

### Economic Analysis

For the 8-yr collection period, backward-looking enterprise budgeting techniques were used for the cow-calf operation to calculate expected annual net returns per cow (AAEA, 2000). Cows were placed into tiers depending on their body weight each year, using 22.67 kg increments beginning at <430.84 kg and the last tier being >634.92 kg for analysis (similar to Bir et al., 2018) resulting in 11 enterprise budgets per year. All prices and costs described below are only those belonging to the cow-calf enterprise on the farm.

Expected annual return per cow is calculated as:

Expected net return ($/cow)=Expectedrevenue(fixedcosts+variablecosts)  (2)

Revenue was calculated for three sources: weaned calf, cull cow sales, and cull bull sales:

$$\text{Expected Revenue }\left(\$/\text{cow}\right)=\text{SteerRev}.+(\text{HeiferRev}.\times 0.\text{6793})\times \text{Prob}\left(\text{Cull}|{\text{Age}}_{\text{t}}\right)+\text{CullCowRev}.\times \text{Prob}\left(\text{Cull}|{\text{Age}}_{\text{t}}\right)+\text{CullBullRev}.\times \left(\text{1}/\text{125}\right)\;$$ (3)

where Price_t_ is the cull cow price in year *t*, and Prob(Cull|Age_t_) is the probability of the cow being culled at age *t*.

Prices for steers and heifers were obtained from Livestock Marketing Information Center using Iowa market data for medium and large frame #1–2 (LMIC, 2019b). Heifer sales were adjusted using a 32.07% retention rate that was recorded on farm over the study period. This high retention rate was due to the management program for first calf heifers to breeding during a short window (~4 wk). Cull cow and bull prices were based on Lancaster, Pennsylvania sale prices (LMIC, 2019c). Following Azzam et al. (1990), cull cow revenue was calculated as:

$$\begin{array}{l} Cull\;cow\;revenue=Cow\;weight\times Price_{t}\\ \;\times Prob\left(Cull|Age_{t}\right) \end{array}$$ (4)

Variable costs included feed cost, marketing costs, mineral, veterinary costs, equipment/facility repairs, labor, interest on operating capital, and other variable costs. Cattle only received supplement in the form of hay during times of limiting forage growth and winter, feed cost was calculated as:

Feed cost = [Land ($/ha)×stockingrate(ha/cow)]+[Annualhayintakepercow(kghay/cow)×Hayprice($/kghay)]  (5)

Forage and hay consumption were calculated as described in the previous section. Annual land cost varied by year, based on USDA NASS published land rental prices (USDA-NASS, 2019c). Hay cost was represented by August hay prices in the United States from 2011 to 2018 sourced from the Livestock Marketing Information Center (LMIC, 2019a). Marketing cost was calculated to be 5% of annual revenue, and mineral cost was estimated to be $36 per cow in 2019 based on farm consumption rates. This cost was then adjusted back to 2011 prices using index for prices paid published by the USDA NASS (USDA-NASS, 2019a). Yearly veterinary costs and equipment/facility repairs were calculated similarly, with 2019 prices estimated to be $25 per cow and $7.87 per ha, respectively. Labor was calculated using hourly wage rates from 2011 to 2018 published by the USDA NASS for 500 h of required labor (USDA-NASS, 2019b). Fuel and other costs were calculated to be $17.20 per ha on farm in 2014 and was adjusted using the prices paid index (USDA-NASS, 2019a). Interest on operating capital was assumed to be 5% of costs annually.

Fixed costs included pasture care, taxes at 3.3% of expenditures, machinery and livestock costs, and miscellaneous cost (10% of overhead cost). Annual machinery depreciation costs for: one 130-hp tractor, one 2011 John Deere Gator, 4 hay rings, a bale unroller, $15,000 barn, chutes/pens, and headgate were calculated using straight-line depreciation. Useful life for machinery was assumed to be 10 yr with the exception of the barn and chute/pens which had estimated useful years of 30 and 20 yr, respectively. Pasture care was comprised of fence, water, seeding (over 30 yr) and lime application every 10 yr and cost $26.04 annually. Straight-line depreciation was used to calculate fixed costs of bulls and cows. Bull weight was estimated by dividing cow weight by 0.70, to service 25 cows and have a 5-yr service period, with an initial purchase price of $3,060 and average price of cull bulls was used to calculate annual depreciation (Bir et al., 2018). Cow depreciation cost was calculated with an initial purchase price of $1,200 and salvage value of $782.65 after 10 yr, using the average weight of the cow herd as the average cull weight for the herd.

Expenses are detailed for each year in Table 2.

**Table 2.** Expenses and budget assumptions for the model beef cow herd, 2011–2018

Expenses	2011	2012	2013	2014	2015	2016	2017	2018
Variable costs								
Marketing, 5% of revenue	5%	5%	5%	5%	5%	5%	5%	5%
Feed, $/ha	31.72	61.72	64.19	74.07	74.07	74.07	69.14	74.07
Hay, $/ton	196.00	203.00	199.00	207.00	161.00	137.00	147.00	177.00
Mineral, $/cow	32.72	34.16	34.79	36.65	36.23	34.65	34.88	36.00
Veterinary, medicine, and identification, $/cow	22.73	23.72	24.16	25.45	25.16	24.07	24.22	25.00
Equipment, and facility repairs, $/ha	7.22	7.53	7.67	8.20	7.99	7.64	7.69	7.87
Labor, $/cow	30.75	31.91	32.97	33.53	34.83	36.06	37.00	39.36
Other variable costs (fuel etc.), $/ha	14.96	15.62	15.90	17.00	16.56	15.84	15.95	16.32
Interest on operating capital^*a*^	5%	5%	5%	5%	5%	5%	5%	5%
Fixed costs								
Taxes^*b*^	3.30%	3.30%	3.30%	3.30%	3.30%	3.30%	3.30%	3.30%
Pasture care, $/cow	26.04	26.04	26.04	26.04	26.04	26.04	26.04	26.04
Machinery, Livestock, Interest, $/cow	28.06	28.06	28.06	28.06	28.06	28.06	28.06	28.06
Miscellaneous, 10% of overhead	10%	10%	10%	10%	10%	10%	10%	10%

^*a*^Interest on operating capital is 5% of variable costs.

^*b*^Taxes represent 3.3% of total expenditures.

### Statistical Analysis

Biological efficiency data for the entire herd were analyzed using a linear mixed effects model in R (R Core Team, 2019, v. 3.6.1; *N* = 1,038). Fixed effects were cow body weights adjusted to BCS of 5 as a continuous variable, cow age and sex of the offspring. Year and cow were included as random terms in the final model. Model fit was tested with random terms included as nested or crossed effects using log-likelihood and final models included both year and cow as crossed random terms (Bates, 2010). Dependent variables of interest were adjusted 205 d calf WW as a percent of BCS adjusted DBW, adjusted 205 d WW and YW. Quadratic terms for age and the natural log of cow body weight were tested for significance and dropped until model fit was not improved based on log-likelihood.

Output per ha (WW/ha) was analyzed via linear regression using adjusted cow body weight and year as explanatory variables. Expected net returns per cow and ha from 2011 to 2018 was analyzed by separating cows into eleven 22.67 kg weight classes, with class 1 being < 430.84 kg and class 11 being > 634.92 kg and used as an explanatory variable with year. Significant differences are declared at *P* ≤ 0.05 and tendencies at 0.05 < *P* ≤ 0.10.

### Net Present Value

Net present value, a measurement of the value of future cash flows over the lifecycle of the cow, was calculated with increasing cow weights to determine which cow size provided maximum present value over their productive lifetime (Moss et al., 2018). Using the enterprise budgets described above, and the biological efficiency and WW models developed in this paper, NPV was forecasted over 10-yr production period similar to Bir et al. (2018) as:

$$\begin{array}{l} NPV=\;NPV_{i}|CowWght_{it}\sum^{10}[Expected\;NetRet_{t}|\\ \;CowWght_{it}/\left({\left(1\;+\;0.05\right)}^{t}\times \;Ha\;per\;cow\right)] \end{array}$$ (6)

where *i* is the *i*th cow in year *t* and a 5% discount rate. Expenses for 2019–2027 are detailed in Table 3, and the size of the operation was assumed to be 40.5 ha with herd sizes determined by the forage intake and land use developed in this paper. Historical prices from the 2004 to 2014 cattle cycle were used to forecast cattle prices and hay prices from 2020 to 2027 (LMIC, 2019b). Future prices were then calculated by the percentage change between years with actual 2019 prices used for the first year of the forecasting model (Bir et al., 2018).

**Table 3.** Expenses and budget projections for the model beef cow herd, 2019–2027

Expenses	2019	2020	2021	2022	2023	2024	2025	2026	2027
Variable costs									
Marketing, 5% of revenue	5%	5%	5%	5%	5%	5%	5%	5%	5%
Feed, $/ha	69.13	69.13	69.13	69.13	69.13	69.13	69.13	69.13	69.13
Hay, $/ton	192.51	194.29	240.63	319.06	194.29	210.33	349.37	361.84	354.71
Mineral, $/cow	36.00	36.36	36.72	37.09	37.46	37.84	38.21	38.60	38.98
Veterinary, medicine, and identification, $/cow	25.38	25.63	25.89	26.15	26.41	26.67	26.94	27.21	27.48
Equipment, and facility repairs, $/ha	7.77	7.84	7.92	8.00	8.08	8.16	8.24	8.33	8.41
Labor, Wage, $/hour	14.61	15.05	15.49	15.93	16.37	16.81	17.25	17.69	18.16
Other variable costs (fuel, etc.), $/ha	16.48	16.65	16.81	16.98	17.15	17.32	17.50	17.67	17.85
Interest on operating capital^*a*^	5%	5%	5%	5%	5%	5%	5%	5%	5%
Fixed costs									
Taxes^*b*^, $/cow	2.80%	2.80%	2.80%	2.80%	2.80%	2.80%	2.80%	2.80%	2.80%
Pasture care, $/ha	24.49	24.49	24.49	24.49	24.49	24.49	24.49	24.49	24.29
Machinery, Livestock, Interest^*c*^, $/cow	93.56–95.00	—	—	—	—	—	—	—	—
Miscellaneous, 10% of overhead	10%	10%	10%	10%	10%	10%	10%	10%	10%

^*a*^Interest on operating capital is 5% of variable costs.

^*b*^Taxes represent 2.8% of total expenditures.

^*c*^Varies by cow weight and herd size.

Land rental prices changed little for this region over time and were assumed to stay static at $69.13 per ha. Hay prices were forecasted using percentage change between years of historical prices from 2004 to 2014. A 1% annual inflation was applied to mineral, veterinary, equipment/facility repairs, and other variable costs (USDA-NASS, 2019a). Labor rates were forecasted using percentage change from 2011 to 2018 rates. Assumption regarding marketing costs and interest on operating capital remained the same as the 2011 to 2018 enterprise budgets. Fixed costs were calculated the same as described above but with prices adjusted to reflect 2019 tax rates, equipment costs, and livestock prices. Pasture care costs stayed static at $24.49/ha and machinery/livestock depreciation ranged from $93.56 to $95.00/cow, varying by cull cow weight and herd size for each simulated weight class. The tax rate was a constant 2.8% and miscellaneous cost remained 10% of overhead costs.

Net present values were then calculated for cows weighing 430.84 kg, then increasing in increments of 22.68 kg up to a final weight of 634.92 kg. Cows were projected to reach 85% of their mature weight by age 2, and increase in weight annually by 4% of their mature weight until reaching maximum weight at age 6 (Selk, 2005). The baseline scenario was assumed to have 200 grazing days and 164 d of hay intake, with hay intake being a combination of 49 d without cold stress and 115 d with cold stress (usclimatedata.com). A sensitivity analysis was then performed by altering grazing day and hay intake without cold stress in 5 d increments above and below the 200 d baseline. The tested range was 175 to 225 grazing days and 139 to 189 hay intake days, when grazing day increased hay intake day decreased and vice versa. When hay feeding days were increased, daily consumption was calculated using the pregnant lactation intake equation.

## RESULTS AND DISCUSSION

### Herd Metrics

Data used for the development of the biological efficiency models and analysis of economic returns were collected from the Lake City AgBioResearch center cow herd from 2011 to 2018. Mean cow age over the course of the recorded period was 4.57 ± 2.11 yr with an averaged nonadjusted BW of 546.11 ± 69.25 kg. Body condition score adjusted cow BW averaged 538.74 ± 65.37 kg (Table 4), with an average BCS of 5.37 ± 0.44 at weaning over the 8-yr time period. As cows aged, their body weight increased in a sigmoidal curve with the inflection point of the line at 534.70 kg which occurred at approximately 4 yr of age.

**Table 4.** Cow-calf herd summary statistics, 2011–2018

Total records	1,038	Min	Max
Average cow weight, kg	538.74 ± 65.37	367.35	780.05
Average BCS	5.37 ± 0.44	4	7
Recorded weaning weights	1035		
Overall average weight, kg	242.65 ± 31.77	108.84	312.93
*Female records*	472		
Average weight, kg	234.65 ± 29.02	108.84	287.98
*Male records*	563		
Average weight, kg	249.35 ± 32.45	122.49	312.93
Recorded yearling weights	868		
Overall average weight, kg	327.04 ± 46.17	185.94	512.47
*Females records*	394		
Average weight, kg	299.63 ± 33.39	185.94	394.55
*Males records*	474		
Average weight, kg	349.83 ± 42.64	231.29	512.47

There were a total of 1,035 calves with recorded WW, with an average of 242.65 ± 31.77 kg (Table 4). Females averaged 14.7 kg less than the male counterparts (234.65 vs. 249.35 kg, females and males, respectively) with 472 recorded female weights and 563 recorded male offspring. These WW were similar to nationwide survey data collected by NAHMS (2009) who reported average WW to be 240.36 kg. Yearling weights were recorded for 868 offspring with an average weight of 327.05 ± 46.17 kg (Table 4). Similar to WW, female offspring weighed less than males at 1 yr of age, with females weighing 49.66 kg less (299.64 vs. 349.83 kg, females and males, respectively) and consisted of a total of 394 female and 474 male records.

### Biological Efficiency Models and Cow Longevity

Calf WW as a percent of cow body weight averaged 45.53% over the study period. The best fit model for CWP included log-transformed DBW, calf birth weight, sex, age of cow, and age of cow squared. As DBW increased, the percentage of their body weight weaned decreased significantly (Table 5; *P*< 0.001). For every 1% increase in body weight, CWP was reduced by 0.38% (−38.58 × ln(DBW) ± 1.87). Sex had a significant relationship with CWP (*P* < 0.01) with female calves having a lower weaning percentage than steers. Age of the cow had a tendency for a quadratic relationship with CWP, where older cows weaned more than younger cows, but at a diminishing rate (*P* = 0.06). This quadratic relationship was similar to the weaning-weight model developed by Bir et al. (2018).

**Table 5.** Regression coefficients for predicting calf weaning weight as percent of cow body weight

Predictors	Estimates	95% CI	*P*
Intercept	277.52	253.78 to 301.26	<0.001
Ln(DBW), kg^*a*^	−38.58	−42.25 to −34.91	<0.001
Calf birth weight, kg^*b*^	0.07	0.02 to 0.13	0.009
Heifers	2.93	−6.38 to 12.25	0.537
Steers	5.95	−3.36 to 15.27	0.211
Age, years	0.98	0.29 to 1.67	0.005
Age squared, years	−0.06	−0.12 to −0.01	0.019
Observations	1,013		
Marginal *R*^2^/conditional *R*^2^	0.380/0.615		

^*a*^Natural log of normalized cow body weight.

^*b*^205 d adjusted weaning weight.

These results were similar to those of Scasta et al. (2015), who separated cows into four BW tiers by 45.5 kg increments: 453, 544, 589, and 634 kg. They found that as cow size increased CWP decreased and reported similar mean efficiencies to those found in the current study. However, their study was conducted in a lower rainfall area where lighter cows were expected to have an energetic advantage. In a study in southwest Arkansas, Beck et al. (2016) showed that although WW increased as cow BW increased, weaning efficiency decreased linearly by 6.7 kg per 100 kg of cow BW. In this study, for every 1% increase in DBW, WW increased 0.36 kg but was offset by the decreased weaning efficiency of 0.38% DBW (Tables 5 and 6; *P* < 0.01). Williams et al. (2018) reported that for cows classified as having high WW ratios, lighter weight cows had higher CWP than heavier cows. However, cows classified as high CWP did consume more feed on a g/kg BW basis.

**Table 6.** Regression coefficients for predicting 205 d adjusted weaning weight

Predictors	Estimates	95% CI	*P*
Intercept	−47.07	−171.74 to 77.61	0.460
Ln(DBW), kg^*a*^	36.92	17.61 to 56.22	<0.001
Calf birth weight, kg^*b*^	0.37	0.10 to 0.65	0.008
Heifers	17.73	−30.96 to 66.42	0.476
Steers	33.79	−14.89 to 82.47	0.174
Age, years	5.80	2.19 to 9.41	0.002
Age squared, years	−0.41	−0.69 to −0.13	0.005
Observations	1,013		
Marginal *R*^2^/conditional *R*^2^	0.119/0.463		

^*a*^Natural log of normalized cow body weight.

^*b*^205 d adjusted weaning weight.

Age of cow had a quadratic relationship with 205 d adjusted WW, similar to CWP (Table 5; *P* < 0.01). Age of cow may influence WW and CWP due to a higher maintenance energy requirement of younger cows. Wiseman et al. (2019) reported maintenance energy requirement of 107 kcal ME/kg BW^0.75^ for primiparous Angus cows in Oklahoma, although the relationship between age and maintenance requirements is inconsistent in the literature (NASEM, 2016). Additionally, the effect of age on milking performance could play a role on WW. Andersen et al. (2020) reported a significant difference in milking output between young and mature Hereford × Angus crosses and Angus cows, with young cows averaging 6.6 kg/d and mature cows producing 8.2 kg/d, although the impact of milk productivity on calf WW is inconsistent (Mulliniks et al., 2020).

For YW, the best fit model included Ln(DBW), calf birth weight, sex, age of cow, and age squared (Table 7). Log-transformed DBW did not have a relationship with YW (*P* = 0.26), unlike WW. Age of dam did have a quadratic relationship with YW (*P* < 0.01). Recent literature has not examined the impact of age on YW, but Koch and Clark (1955) reported that dam age impacted YW and was similar to WW, as reported here, but was likely not practically important. When expressed as a percentage of DBW, there was a significant relationship and resulted in a depression in YW efficiency by 0.58% for each additional 1% increase in body weight (*P* < 0.001), similar to the results with weaning efficiency. This is similar to Morris and Wilton (1976) who reported that both weaning and YW efficiencies were superior for smaller cows. It should be mentioned, however, that these animals were in a grass-finishing system and results may be different from grain finishing systems.

**Table 7.** Regression coefficients for predicting calf yearling weight

Predictors	Estimates	95% CI	*P*
Intercept	155.72	−38.82 to 350.26	0.117
Ln(DBW), kg^*a*^	17.46	−12.99 to 47.91	0.262
Calf birth weight, kg^*b*^	0.37	−0.08 to 0.81	0.11
Heifers	−5.23	−77.37 to 66.91	0.887
Steers	45.00	−27.12 to 117.12	0.222
Age, years	9.19	3.55 to 14.82	0.001
Age squared, years	−0.65	−1.09 to 0.020	0.004
Observations	847		
Marginal *R*^2^/conditional *R*^2^	0.314/0.427		

^*a*^Natural log of normalized cow body weight.

^*b*^205 d adjusted weaning weight.

The results of Mulliniks et al. (2018) highlight the flaws of using biological efficiency alone as a selection metric. Reproduction performance was reported to decrease with decreasing cow body size with a 435.83 kg cow having a pregnancy rate of just 86% compared to 97% with a 538.32 kg cow (Mulliniks et al., 2018). Mulliniks et al. (2018) hypothesized that the reduction in pregnancy rate may be due to an imbalance between genetic potential for milk production and forage intake with smaller cows unable to consume enough forage to meet their nutritional demand for lactation. This goes against Stewart and Martin (1981), who reported that lifetime performance of Angus and Milking Shorthorn influenced cattle decreased with increasing mature body weight by −0.007 ± 0.003 calves/kg. However, we did not have records on culling decisions and therefore could not accurately analyze the impact of cow size on longevity. Additional research in this area is needed as breed differences have been reported in previous literature (Tanida et al., 1988; Núñez-Dominguez et al., 1991; Szabó and Dákay, 2009).

### Forage Intake and Land Use

**Figure 1.**
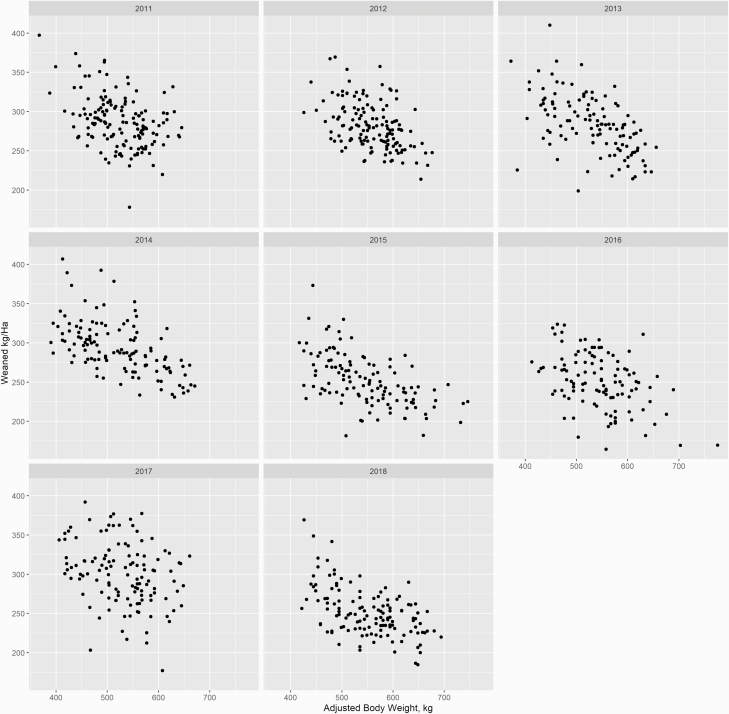
Weaned kg/ha plotted against adjusted cow body weight, by year for model herd.

As cow size increased, forage consumption increased 1.69 kg of DM/d for each additional 100 kg of body weight. This resulted in 450 kg cows consuming approximately 10.84 kg of DM/d and 750 kg cows consuming approximately 15.91 kg of DM/d during the grazing period. Over a 200 d grazing season, this resulted in 750 kg cows consuming 1,014.3 kg of DM more than 450 kg cows (3,181.85 kg of DM vs. 2,167.55 kg of DM, respectively). Over the record period forage productivity ranged from 5,000 kg of DM/ha to 6,587 kg of DM/ha and had an average of 5,662 kg of DM/ha from 2014 to 2018. Forage productivity for 2019 was included in calculating the average forage productivity applied for the years of 2011 to 2013 which was 5,506 kg of DM/ha. The long-term estimated utilization was 50% and resulted in stocking rates of 0.77 ha/cow for 450 kg cows up to 1.15 ha/cow for a 750 kg cow. Winter-feeding rates followed similar trends. Daily hay intake increased across body weights at a rate of 1.59 kg per 100 kg increase in body weight and resulted in total feeding rates of 1,636.52 kg of DM and 2,418.03 kg of DM for 450 and 750 kg cows, respectively. Stokes et al. (1986) simulating the changes in stocking rate with increasing cow size in central Texas on improved forages with a 50% utilization rate and reported a stocking rate of 0.76 ha/cow for a moderate milking 550 kg cow versus 0.66 ha/cow for a moderate milking 450 kg cow. Lindquist (2014) reported that for Michigan cow-calf operations stocking rates ranged from 1.01 to 1.83 ha/cow, similar to the range calculated here. The stocking rates changed by year according to forage productivity and each year’s rates were utilized for the economic analysis. These results led to lighter cows weaning more kg/ha than heavier weight cows, with a decrease in 26.38 kg/ha with every 100 kg increase in body weight with year included in the model (Fig. 1; *P* < 0.01; *R*^2^ = 0.41). Bir et al. (2018) reported a similar advantage for lighter weight cows weaning more weight per ha. This would indicate that in the Upper Midwest, producer adoption of lighter cows may be an option for meeting the goals of sustainable intensification by maximizing productivity per unit land (Makkar, 2013; Tedeschi et al., 2015).

### Lake City Expected Net Return 2011–2018

The expected net returns of the Lake City cow herd from 2011 to 2018 was analyzed with cow body weight classification (1 to 11 in 22.67 kg increments) and year used as explanatory variables and individual cow as a random term. Overall, mean returns were $338.97 from 2011 to 2018 per cow per year. Net returns had a significant relationship with year, with highest returns over the trial period in 2014 and 2015 (*P* < 0.01). Both years corresponded with the highest calf prices over this time and average years of forage productivity (5,398.16 and 5,740.53 kg/ha for 2014 and 2015, respectively). Lowest returns occurred in 2016 with a decrease in $88.21 on the intercept of the regression line, due to a depressed weaned calf price that fall.

**Figure 2.**
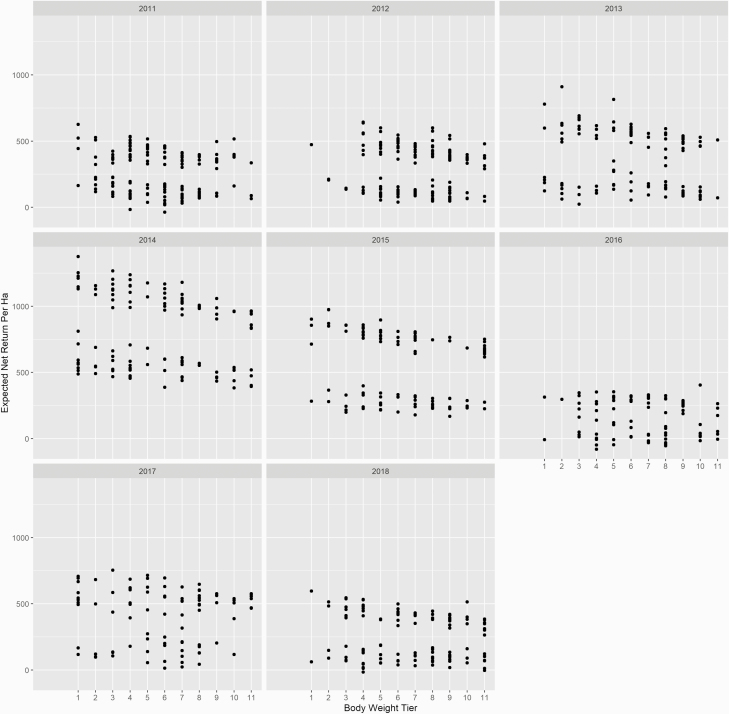
Expected net return/ha plotted against 11 cow body weight tiers (22.67 kg intervals) beginning at 430.83 kg.

Cow body weight classification did not have a significant relationship with expected net returns per cow (*P* = 0.19). These results are different from those reported by Doye and Lalman (2011), who reported that as cows increased in size their expected net returns decreased. The returns in this study reflect that the forage base on the farm was not limiting economic returns of heavier cows on a per cow basis at 200 grazing days, and that cows over this period were generally well matched to the forage base and management system with lighter cows offsetting their increased variable costs and heavier cows offsetting their increased feed costs equally on a per cow basis. On a per ha basis, cow body weight classification did have a significant relationship with expected net returns (*P* < 0.01; Fig. 2). Expected net returns per ha decreased $10.27 with each additional body weight classification. Similarly, Beck et al. (2016) reported that cow BW did not impact net returns, but increased stocking rate increased net returns by $438/ha. The results are reflective of the increased stocking rate potential and output per hectare with lighter cows over the study period.

### Net Present Value

Net present value over 10 yr was calculated for cows beginning at 430.84 kg and 22.67 kg increments to a maximum weight of 634.9 kg (Tables 7 and 8). Weaning weights were calculated using the equation reported in Table 5. The baseline scenario assumed 200 grazing days and 164 hay feeding days, and the maximum NPV was determined. At the baseline, cows weighing 453.51 kg had the maximum NPV at ($496.63), followed by the 476.19 kg classification, the 430.84 kg classification and then decreasing in value with each additional 22.67 kg (Table 8). Ultimately, the 634.92 kg classification had approximately 74% lower NPV compared to the 453.51 kg classification at 200 d grazing. These results were similar to the NPV result of Bir et al. (2018) who found that lighter cows had higher NPV than their heavier counterparts. Nasca et al. (2015), in an economic and environmental analysis of beef production in Argentina, reported economic efficiency declined with increasing cow weights because of high supplementary feed costs, similar to this study.

**Table 8.** Net present value at increasing cow body weight and variable grazing days^a^

Cow (kg)	Grazing days										
	175	180	185	190	195	Baseline	205	210	215	220	225
430.84	−$585.56	−$561.44	−$537.32	−$513.20	−$489.08	−$537.44	−$443.04	−$421.11	−$399.18	−$377.26	−$355.33
453.51	−$618.96	−$594.85	−$570.75	−$546.64	−$522.54	−$496.63	−$476.45	−$454.46	−$432.47	−$410.48	−$388.49
476.19	−$654.92	−$630.83	−$606.74	−$582.65	−$558.56	−$534.46	−$512.42	−$490.37	−$468.33	−$446.28	−$424.23
498.87	−$670.98	−$646.90	−$622.82	−$598.74	−$574.67	−$550.59	−$528.49	−$506.39	−$484.29	−$462.19	−$440.09
521.54	−$687.17	−$663.10	−$639.03	−$614.97	−$590.90	−$566.84	−$544.69	−$522.54	−$500.39	−$478.25	−$456.10
544.22	−$722.34	−$698.29	−$674.23	−$650.18	−$626.12	−$602.07	−$579.87	−$557.68	−$535.49	−$513.29	−$491.10
566.89	−$739.43	−$715.39	−$691.34	−$667.30	−$643.25	−$619.21	−$596.97	−$574.73	−$552.50	−$530.26	−$508.03
589.57	−$756.81	−$732.78	−$708.74	−$684.71	−$660.67	−$636.64	−$614.36	−$592.08	−$569.81	−$547.53	−$525.26
612.24	−$774.59	−$750.56	−$726.53	−$702.51	−$678.48	−$654.45	−$632.14	−$609.83	−$587.51	−$565.20	−$542.89
634.92	−$792.81	−$768.80	−$744.78	−$720.76	−$696.74	−$672.72	−$650.37	−$628.03	−$605.68	−$583.33	−$560.98

^*a*^Baseline= 200 d grazing.

The ideal cow weight in this study changed when a sensitivity analysis on the number of grazing days was performed (Tables 8 and 9). When grazing day was increased 5 d, the 430.84 kg classification had the highest returns, and this held true for the rest of the grazing day scenarios. Cows weighing 566.89 kg and higher never provided higher returns than the 453.51 kg classification at 200 d grazing, even at 225 grazing days. Interestingly, when grazing days were reduced, the 430.84 kg classification had higher returns compared to their baseline, before ultimately dropping below the baseline value at 180 d grazing. This shows that lighter cows can withstand a drop in grazing days because hay costs are not as high compared to heavier cows, which may provide producers some protection against adverse weather conditions. Scasta et al. (2015) reported similar results when comparing drought performance of small and large cows. They found that large cows did not maximize their genetic potential during years of drought whereas smaller cows had an advantage because of their lower maintenance energy requirements. Therefore, at 200 d grazing, heavier cows (>476.19 kg) provided increasingly less NPV than the three lightest cow weigh classes with 453.51 kg classification providing the highest return. Additionally, the lightest cows (430.84 kg) increased their NPV through 180 grazing days. This indicates that for the Upper Midwest, light cows are more functional for producers as they provide protection against adverse climatic events, which may reduce the number of days on pasture.

**Table 9.** Relative change (%) in net present value compared to a 430.84 kg cow at 200 grazing days^*a,b*^

Cow BW (kg)	Grazing days										
	175	180	185	190	195	Baseline	205	210	215	220	225
430.84	−8.95%	−4.47%	0.02%	4.51%	9.00%	0.00%	17.56%	21.64%	25.72%	29.80%	33.88%
453.51	−15.17%	−10.68%	−6.20%	−1.71%	2.77%	7.59%	11.35%	15.44%	19.53%	23.62%	27.71%
476.19	−21.86%	−17.38%	−12.89%	−8.41%	−3.93%	0.55%	4.66%	8.76%	12.86%	16.96%	21.06%
498.87	−24.85%	−20.37%	−15.89%	−11.41%	−6.93%	−2.45%	1.66%	5.78%	9.89%	14.00%	18.11%
521.54	−27.86%	−23.38%	−18.90%	−14.43%	−9.95%	−5.47%	−1.35%	2.77%	6.89%	11.01%	15.13%
544.22	−34.41%	−29.93%	−25.45%	−20.98%	−16.50%	−12.03%	−7.90%	−3.77%	0.36%	4.49%	8.62%
566.89	−37.58%	−33.11%	−28.64%	−24.16%	−19.69%	−15.21%	−11.08%	−6.94%	−2.80%	1.33%	5.47%
589.57	−40.82%	−36.35%	−31.87%	−27.40%	−22.93%	−18.46%	−14.31%	−10.17%	−6.02%	−1.88%	2.27%
612.24	−44.13%	−39.66%	−35.18%	−30.71%	−26.24%	−21.77%	−17.62%	−13.47%	−9.32%	−5.17%	−1.01%
634.92	−47.52%	−43.05%	−38.58%	−34.11%	−29.64%	−25.17%	−21.01%	−16.86%	−12.70%	−8.54%	−4.38%

^*a*^Baseline= 200 d grazing.

^*b*^Positive number reflects positive change in net present value compared to a 430.84 kg cow at the baseline.

## CONCLUSION

Cow-calf production systems are highly variable and balancing cow size with both the management and grazing environment may help improve the system profitability (Nasca et al., 2015). These results indicate that in the Upper Midwest utilization of lighter weight cows increases the WW ratio of the herd, may require less land and hay per cow, and potentially increases expected net returns on a per ha basis. The NPV of light weight cows increased as the number of grazing days decreased, as they require less hay compared to their heavier counterparts. This may provide protection for producers against adverse weather events and climatic variability that is predicted to increase in frequency in Michigan (Melillo et al., 2014). Additionally, the increased weaned weight per ha captured by the lighter cows may also meet the goals of sustainable intensification by maximizing production per unit of land (Makkar, 2013; Tedeschi et al., 2015). This would require a paradigm shift from producers, which often believe that heavier cows maximize profitability and require improved estimates of cow size from producers, as most do not weigh their animals consistently (Doye and Lalman, 2011; Reuter, 2017). Research on other regions, such as that done by Nasca et al. (2015) and Bir et al. (2018), should be done in other regions to provide recommendations for producers in those regions on how best to maximize productivity. This study did not include actual animal intakes and milk production, factors that have been reported to impact calf WW and differ for cows with high CWP (Williams et al., 2018). These results do not, however, equate to what is profitable for packer and feedlot operators in the region, as what is profitable for cow-calf producers may not be as profitable for other market segments. There may be potential for collaboration between feedlot and cow-calf sectors, as research has shown that in some regions offspring of small and moderate frame cows were more profitable and had higher BW in the feedlot (Mulliniks et al., 2018). To our knowledge this is the first work to show the relationship between biological efficiency, economic returns, and beef cow body weight in the Upper Midwest region of the United States. The biological efficiency advantage reported here in light weight cows needs continued research by measuring on farm intakes, milk production, and examining how selection for lighter weight cows alters economic returns further up the beef supply chain.

## Footnotes

^1^ Forage and hay core samples were not taken in other years.
